# Factors Associated with Accessing Prison Dental Services in Scotland: A Cross-Sectional Study

**DOI:** 10.3390/dj7010012

**Published:** 2019-02-01

**Authors:** Ruth Freeman, Derek Richards

**Affiliations:** 1 Dental Health Services Research Unit, University of Dundee, Dundee DD1 4HN, UK; 2 Public Health, NHS Tayside, Dundee, DD2 1UB, UK; 3 Centre for Evidence-based Dentistry, DHSRU, University of Dundee, Dundee DD1 4HN, UK; derek.richards@nhs.net; 4 Dental Public Health South East Scotland, NHS Forth Valley, Stirling FK9 4SW, UK

**Keywords:** prison, accessible dental services, oral health-related quality of life, obvious decay

## Abstract

**Background:** Prisoners have poorer dental health than non-prison populations. It is known that the prison environment can promote health and thus, policies, including access to dental care, are in place to promote health during imprisonment. **Aim:** Our aim was to conduct an oral health and psychosocial needs survey to identify the factors associated with accessing prison dental services in Scotland. **Methods:** A convenience sample of offenders from a male maximum security prison, a women’s prison, and a young offenders’ institution was gathered. A questionnaire examined the demography, prison experience, dental anxiety, oral health-related quality of life, and reported attendance of dental services. A dental examination was conducted using the International Caries Detection and Assessment System to diagnose obvious decay. A hierarchical logistic regression analysis was performed. **Results:** 342 prisoners participated. When missing data were excluded, the final sample was 259. The regression analysis showed the following: Model 1 characterized the offenders by demography and prison experience, explaining 19% of the variance. Model 2 showed that an offender was 36% more likely to attend dental services for every unit change in the 5-point ranking scale of ‘feeling irritable with people because of teeth, mouth, or dentures’, explaining an additional 7% of the variance. Model 3 explained 35% of the variance, (i.e., an additional 9%) and was adopted as the final model to characterize offenders who access dental services when in prison. An offender who reported accessing prison dental services was 3.28 times more likely to be male. For each increase in the year of an offender’s age, the offender was 5% more likely to access prison dental services. An 11% greater chance of accessing prison dental services for every experience of remand was also found. An offender was 32% more likely to access prison dental services for each increased level of irritability, and there was a 2 times higher likelihood of emergency dental services’ attendance. There was a 19% lower chance of accessing prison dental services for each additional tooth affected by decay and a 13% greater chance of accessing prison dental services for each unit increase in missing teeth. **Conclusions:** In conclusion, this investigation identified factors associated with access to prison dental services in Scotland. The role of accessibility factors, such as the oral health impact of irritability, appeared to increase perceptions of dental need and promote dental services’ attendance.

## 1. Background

Prisoners have greater numbers of decayed and missing teeth but fewer filled teeth when compared with the non-prison population [1,2,3,4,5,6,7]. While lifestyle issues prior to imprisonment are important, the prison environment with its routines and structures can promote oral health while also having the potential to exacerbate unhealthy behaviors [2,5,6]. Consequently, the concept of the health-promoting prison has moved to center stage, ensuring that policies have been put in place to promote health within the prison environment [8,9,10]. The promotion of oral health, for instance, is a central strand of the World Health Organization’s European policy for offenders and ex-offenders. Attendance of dental care services has been linked to improved self-care and quality of life for offenders and ex-offenders [8,9,10].

Current research reports that people in prison, even when they have toothache [1,2,3,5,6,7], experience difficulties when trying to access dental services [5]. It would seem appropriate, therefore, to investigate the background factors related to the utilization of prison dental services. The difficulties people in prison encounter when accessing dental services are stated to be associated with poor literacy, poor health literacy, dental fear, and low perception of need [5,11,12,13,14,15]. These so-called ‘patient factors’ are reflected in Cohen’s [16] accessibility framework. However, the observations that 23% of offenders reported that they had a pattern of regular dental attendance for examination and routine dental treatment outside of prison and 33% of offenders reported that they have attended examinations and routine dental treatments inside prison [11] have permitted the research questions to be raised about the role of Cohen’s [16] accessibility factors as explanatory elements for offenders when they access dental services inside prison.

Cohen [16], in her seminal paper on accessing dental services, suggested that accessibility factors relating specifically to the patient include dental anxiety, costs of treatment, and perceived need. These factors could either enable or inhibit a person’s access to dental services. Some 30 years later, Marshman et al. [17] revisited the issues of accessibility and showed that, in relation to the patient, the perception of treatment need has remained an important accessibility factor. Focusing on people in prison and their access to prison dental services, Marshman et al. [18] proposed that their perception of dental need, together with their pattern of routine dental attendance outside of prison, has enabled prisoners to engage in routine attendance inside prison. However, the role of perceived need was, according to Marshman et al. [18], found to be poorly associated with accessing prison dental services. Their work [18] seemed to suggest that a person’s pattern of dental services’ attendance outside of prison had a stronger influence upon accessing dental services in prison than their perception of dental need. The research of Marshman et al. [18] thus supported the hypothesis that there must be additional and intervening accessibility factors, such as prison experience, which could influence the utilization of prison dental services. This hypothesis is timely and appropriate, because accessible health services are considered to be of central importance in the promotion of prisoner health [8,9,10]. The following research question remains, however: what are these additional and intervening accessibility factors that affect the utilization of prison dental services during custodial sentences? Therefore, the aim of this study was to conduct an oral health and psychosocial needs survey to identify factors associated with accessing prison dental services in Scotland.

## 2. Methods

### 2.1. Sample

The 3 Scottish prisons were chosen for participation from the prison estate because, [i] they were representative of a maximum security prison for adult male offenders, a prison for women, and female young offenders and a male young offenders’ institution, and [ii] they all had National Health Services dental treatment. The dental treatment provided in the prison setting includes restorative dentistry (conservation and prosthetic and endodontic treatments), the extraction of teeth, preventive dental treatments (e.g., scale and polish), and emergency dental care. The prisoners all had a visiting dentist who provided treatment; however, the availability of such services was affected by security checks, restrictions on movement, finding prison officers to escort prisoners to and from the dental surgery, and competing priorities within the prison environment [19].

A non-probability convenience sample of offenders from these 3 prisons was obtained. A post-hoc power analysis confirmed that a sample size of 250 would give 80% power to detect a one-sided significant increase in the reported prison dental services’ attendance of 9% when the reference category reported a baseline attendance of 45% [20].

The ethical committees required that all the prisoners were to have the same opportunity to participate, and thus, a non-probability sampling technique was appropriate. Offenders, nevertheless, who were assessed by prison staff to pose a risk to the researchers and those who did not understand English were excluded from the survey. Informed consent was obtained from all the prisoners taking part in the survey.

### 2.2. Questionnaire

The questionnaire consisted of the following features.

i. Demographic Profile of the Participants

The first part of the questionnaire gathered information about the participants’ demographic profile, including age in years and gender, previous occupation prior to imprisonment, and prison experience, which included the total length of time in prison, amount of time on remand, and number of prison sentences.

ii. Dental Anxiety Status: The Modified Dental Anxiety Scale (MDAS)

Dental anxiety was assessed using the Modified Dental Anxiety Scale (MDAS). MDAS consists of 5 questions. It asks the participants how anxious they feel regarding waiting for dental treatment, drilling, scaling and polishing, and local anesthesia. The respondents rate their dental anxiety on a 5-point scale, which ranges from not anxious (1) to extremely anxious (5). Possible scores range from 5 to 25, with scores over 19 indicating dental phobia. The normative value for the general population in the United Kingdom is 12.0 [21,22]. The MDAS has good reliability with a Cronbach’s alpha of 0.93 [23]. The Cronbach’s alpha was 0.94 for this sample of people in prison.

iii. Oral Health-Related Quality of Life: The Oral Health Impact Scale-14 (OHIP-14)

The OHIP-14 is a 14-item inventory that assesses oral health-related quality of life. It is based on a hierarchy of impacts arising from oral disease, ranging in severity, and it includes questions on functional limitations (e.g., pronouncing words), physical pain (e.g., painful, aching mouth), psychological discomfort (e.g., feeling self-conscious), physical disability (e.g., interrupted meals), psychological disability (e.g., feeling embarrassed), social disability (e.g., irritable with others), and handicap (e.g., life less satisfying). The respondents were asked how frequently they had experienced each of the oral impacts in the previous 12 months with questions such as ‘Have you had painful aching in your mouth’. The responses were made on a 5-point Likert scale, with scores ranging from 0 (never) to 4 (very often) [24]. OHIP-14 has good reliability with a Cronbach’s alpha of 0.91 [25]. The Cronbach’s alpha for the total OHIP-14 score was 0.95 for this sample of people in prison.

iv. Reported Dental Services’ Attendance Behavior

The final part of the questionnaire asked about access to prison dental services during imprisonment, either for an emergency or a dental examination and routine dental treatments [26]. This was a simple dichotomous variable. In addition, the offenders were asked about their usual pattern of dental services’ attendance outside of prison.

### 2.3. Training for the Administration of the Questionnaire and the Oral Health Examination

Prior to the survey, which took place between September and December 2011, the research team, including the two participating dentists and dental nurses, were trained in the adoption of the operational protocols to gain consent and gather data in the prison setting, as well as breakout training. Breakout training ensured that in the event of a disturbance, the research team would be safe. Training by a health psychologist on how to assist the participants, as required, with the completion of the questionnaire without influencing their responses was also provided.

The dental examiners were specifically chosen, because they had recently been calibrated for a national oral health survey with percentage agreements in the range of 91–100% and a Kappa of >0.8 [27]. They were also chosen because they had experience working in the prison sector. The International Caries Detection and Assessment System (ICDAS) is a clinical, visual scoring system for obvious dental decay [28,29]. In the ICDAS nomenclature, decay is described as D_1_MFT and includes all white spots, brown spots, enamel, and dentine cavitated lesions (ICDAS caries codes 1, 2, 3, 4, 5, or 6). D_2_MFT includes all enamel and dentine cavitated lesions (ICDAS caries codes 3, 4, 5, or 6), and D_3_MFT includes only dentinal cavitated lesions (ICDAS caries codes 3, 4, 5, or 6). ICDAS standardization exercises using the ICDAS criteria for detecting caries [29] were provided by Professor Gail Douglas, the ICDAS coordinator. For ICDAS, the percentage difference in the detection of the category obvious decay (D_3cv_MFT ICDAS caries codes 3–5) between the two dental examiners was 4%, showing a high degree of equivalence (P = 0.34). For the purposes of this oral health needs assessment, ICDAS was used to diagnose obvious decay, and the ICDAS findings were converted to D_3cv_MFT, that is, carious lesions that were dentinal, cavitated (c), and visual (v) [30].

### 2.4. Data Collection Procedure

The participants were asked to complete the questionnaire prior to the dental examination. Assistance was provided to those participants who experienced reading difficulty when completing the survey. This assistance did not influence the participants’ responses, as instructed on the training day. The dental examination took place once the questionnaire was completed. The dental examination was conducted in the prison residential areas with infection control procedures observed. A Daray versatile medical light, as in the National Dental Inspection Programmes [26], was used. The 2 dental nurses assisted with the clinical data collection.

### 2.5. Ethical Issues and Procedures

Ethical approval was obtained from the National Research Ethics Service (reference number NRES 10/S0501/10) and the Scottish Prison Service Ethics Committee. All the data files were held securely on encrypted University computers, and the transcriptions were stored in a secure location. A coding system was used to anonymize the prisoners’ data.

### 2.6. Statistical Analysis

The data were entered into a database and analyzed using SPSS v21 and STAT v13. The data were subjected to frequency distributions, Cronbach’s alpha, chi-squared analyses, and t-tests. A hierarchical multivariable logistic regression analysis was undertaken to characterize the offenders who said they had accessed dental services within the prison estate, either for an emergency or routine dental examination and/or treatment appointment during their imprisonment. The ‘xtmelogit’ procedure was used to enable control of the clustering variable, namely, the prison of confinement. The intra-class correlation coefficient was calculated to determine the level of clustering. The dependent variable was access to dental services when in prison with ‘no’ coded as 0 and ‘yes’ coded as 1. The independent variables were age (in years) and number of prison remands, which were entered into the analysis as Model 1. The remaining independent variables were sequentially included in the analysis as follows: OHIP item ‘feeling irritable with people because of teeth, mouth, or dentures’, pattern of dental attendance outside of prison (emergency = 0: routine = 1), teeth decayed into dentine, and missing teeth.

## 3. Results

### 3.1. The Sample

A convenience sample of 342 prisoners (243 males, 99 females) from the three Scottish prisons participated. All the participants completed the questionnaire, and 87% (208 males, 90 females) had an oral examination. A total of 44 prisoners did not take part in the oral health examination for the following reasons: refusal to be examined (25%), attendance at court (25%), discharged/preparing for discharge from prison (11%), at work/education (14%), moved to another prison (9%), and agency visit (5%). There was no significant difference in the proportion of male and female offenders who participated and did not participate in the oral examination (X^2^[1] = 1.11: P = 0.18). All the missing data were excluded, providing a valid response rate of 76% (259). The statistical analysis was conducted on the 259 complete datasets.

### 3.2. Demographic Profile

The mean age for the participants was 27.21 (±9.80) years. A total of 29% (76) were female. Additionally, 66% (172) were unemployed and not working prior to imprisonment; of the remainder, 75 were in some form of employment, 8 were in training, and 4 were in full-time education. The majority was Caucasian (94%). Over 80% (211) were single.

### 3.3. Prison Experience

The total length of time spent in prison while sentenced ranged from 1 day to 34 years, with an average of 2.02 (±4.14) years. The mean number of remands was 3.84 (±4.93), and the mean number of prison sentences was 2.63 (±3.88). The mean length of time spent in prison, during their current sentence at the time of the survey, was 4.93 (±3.88) months.

### 3.4. Reported Dental Services’ Attendance

A total of 54% (141) of offenders stated that their usual pattern of dental services’ attendance outside of prison was for the relief of pain (emergency care). A total of 46% (118) of participants stated that they had attended the prison dentist during their sentences. Of the 118 prisoners who reported that they had accessed prison dental services, 55% said that they had accessed dental services only in an emergency, and 33% stated that they had attended for a dental examination and routine treatment. The reported treatment received during imprisonment, included restorations (93%); extractions (68%); scaling and polishing (66%); teeth crowns (26%), and treatment for dentures (18%). The offenders who had accessed prison dental services were significantly older than those who had not (t = 4.91: P < 0.001). Prisoners with a significantly greater mean total years of imprisonment (t = 6.24: P <0.001) and those with a significantly greater mean number of times in remand (t = 2.35: P = 0.02) had accessed dental services more often compared with others. For prisoners who had not accessed prisoner dental services, they stated that the barriers to attending prison dental services during imprisonment were difficulty in arranging an appointment (61%), infrequent clinics (48%), and problems getting (10%) and completing (3%) prison request forms.

### 3.5. Oral Health-Related Attitudes

#### 3.5.1. Dental Anxiety

The mean score for dental anxiety was 10.02 (±5.56). There were no significant differences in total mean MDAS scores between those participants who had accessed dental services in the prison setting (10.22 ± 5.59) and those who had not (9.86 ± 5.54) (t = 0.52: P = 0.60).

#### 3.5.2. Oral Health-Related Quality of Life

The mean OHIP-14 score was 15.61 (±14.34) with a range from 0 to 56. There was a significant difference in the total mean OHIP-14 score between those who had accessed dental services in prison (18.37 ± 14.97) and those who had not (13.20 ± 13.35) (t = 2.81: P = 0.005). Table 1 shows the statistically significant differences between the mean scores for the oral health impact items between those who had accessed and those who had not accessed dental services in the prison setting (Table 1). There were no statistically significant differences in mean scores for OHIP items between male, female, and young offenders.

### 3.6. Obvious Decay Experience

Only 10 participants had no obvious signs of dental caries experience. The remainder of the sample had experience of dental caries. For the entire sample (n = 259), the mean number of decayed, missing, and filled teeth (D_3cv_MFT) was 10.21 (±6.32); the mean number of decayed teeth (D_3cv_) was 1.62 (SD ± 2.22); the mean number of missing teeth was 4.36 (±4.45); and the mean number of filled teeth was 4.23 (±3.80). The participants who had accessed dental services in the prison setting had a significantly greater mean D_3cv_MFT, a significantly greater mean number of missing teeth, and a significantly greater mean number of filled teeth (Table 2). More offenders (62%) who had not accessed prison dental services had teeth decayed into dentine than those who had accessed dental services in the prison setting (38%) (X^2^[1] = 5.64: P = 0.02), and the difference was statistically significant.

### 3.7. Identifying Factors Associated with Access to Prison Dental Services 

Table 3 shows the hierarchical multivariable logistic regression analysis, which was conducted to identify factors associated with access to prison dental services. A Poisson regression with a robust variance estimator was also performed, and the results were inspected. No appreciable differences in the substantial effects were found. Hence, the logistic model was selected for ease of presentation. An analysis was conducted to compute the intra-class correlation, which was found to be 0.114: [95 %CI: 0.021, 0.438]: X^2^[1] = 19.33, P < 0.001. Hence, all the regression results presented have been controlled for clustering due to prison membership, preventing biased parameter estimates. It was decided, in addition, to omit gender in the regression, as this was already implicated within the prison category. Age and prison experience were entered into the analysis as Step 1. Model 1 characterized the offenders by demography and prison experience (remand) and explained 19% of the variance. Model 2, while controlling for demography and prison experience, showed that an offender was 36% more likely to attend dental services for every unit change in the 5-point ranking scale of ‘feeling irritable with people because of teeth, mouth, or dentures’ and explained an additional 7% of the variance. Model 3 was adopted as the final model to characterize offenders who accessed dental services when in prison and explained an additional 9% of the variance. Model 3 therefore explained a total of 35% of the variance. Consequently, an offender who accessed dental care during his/her imprisonment had an 8% increased likelihood of attending for every experience of remand, a 32% increased likelihood of accessing dental services for each increased level of irritability, and an over 2 times greater chance of emergency dental services’ attendance. Furthermore, there was an 18% lower chance of accessing dental services for each additional tooth affected by decay, and a 13% greater likelihood of accessing dental services for each unit increase in missing teeth.

## 4. Discussion

The aim of this work was to conduct an oral health and psychosocial needs survey to identify factors associated with accessing prison dental service. The results of the survey identified accessibility factors that characterized the utilization of prison dental services during custodial sentences.

The access of people in custody to prison dental services was the main concern of this study. Because the definition of dental services’ utilization is a combination of associated accessibility factors, it is of importance to identify them and to illustrate how different reasons for accessing dental services may be informative. This study demonstrated that for people in custody, the associated accessibility factors when utilizing dental services were in some ways different to those in the general population [16,17,18]

The predominant accessibility or associated factors that influenced the utilization of prison dental services were age and prison experience (i.e., the number of times in remand). While it is to be expected that longer time in prison would be associated with increased reported access to prison dental services, the mean length of the participating offenders’ current sentences was merely 4.93 months, which was equivalent to the national average sentence length of 4 months [23]. Because attendance could be argued to be a prevalence variable, it may be suggested that an individual whose custodial sentence was 8 months (rather than the 4-month average) would have had twice as much opportunity to access dental care; however, we contend that the above observation indicated that another prison experience factor, in addition to the length of time of the current imprisonment, influenced access to prison dental services. We postulate that because the literature suggests that poor health literacy affects access to health services [12,13,14,15], then an alternative means of gaining information on how to access services must exist. We speculate that increased experience of prison remand could have provided an environment in which the offenders gained information on and knowledge of how to access dental services—whether this was for emergency or routine care [11]. Although oral health-related quality of life differentiated between those who had and had not accessed prison dental services, the same was not so for dental anxiety. Moreover, the mean number of teeth decayed into dentine was 1.62 teeth; however, only 46% had accessed prison dental services—suggesting that this unmet treatment need, as proposed by Marshman et al. [18], had not acted to prompt the offenders to access dental care. Considering that oral health impacts differentiated between those who had and had not accessed prison dental services, we again speculate that when prisoners’ oral health impacts their quality of life, then offenders access prison dental services.

We suggest that the results of the hierarchical multivariable logistic regression analysis support our proposition that an intervening oral health-related quality of life variable increased the offenders’ awareness of their dental needs and enabled access to dental treatment. We propose this because the findings showed that greater experience of prison remand and the impact of oral health upon irritability with others, together with a greater mean number of missing teeth but a lower mean number of decayed teeth, characterized those who accessed prison dental services. The proposition that an association exists between oral health and its impact upon quality of life, which, in turn, raises awareness of perceived need and thus improves access to dental services, is also supported by the work of others [7,14,18]. It is interesting that this specific oral impact—increased irritability with others—appeared to act as a trigger to increase the utilization of prison dental services. This suggestion finds support in the finding that those who had accessed dental services had lower mean scores for irritability. This is the first time that an investigation has shown that the oral health impact of irritability with others (social disability) outweighed dental indifference in a prison population, as reflected in this OHIP item’s ability to increase the explanation of the model. Although this work was conducted in Scottish prisons with a non-probability convenience sample, the finding that the prisoners were irritable with others on account of their teeth is worrisome and should be of interest to anyone working in the prison environment [1,2,3,7,17,18,28].

There are limitations to this work. First, the sample is a non-probability convenience sample, and consequently, there are implications regarding the representativeness of the sample. The mean length of sentence for this sample of prisoners was 4.93 months, which is equivalent to the 4-month Scottish average for a custodial sentence (23); therefore, it may be suggested that, with regard to the average length of sentence, they were equivalent to other prison populations and national averages. In this convenience sample of prisoners, 46% reported they had accessed prison dental services, which approximates to the 43% found by Marshman et al. [18] and the 50% found by Rodrigues et al. [7]. Secondly, this is a cross-sectional survey, and the limitations surrounding the use of such data is acknowledged here, together with the need for additional work to confirm the findings presented here. Therefore, with regard to the reported access to care, this sample was commensurate with others in the United Kingdom and South America. Thirdly, although the dental examiners had been calibrated for a recent national dental survey, they were standardized for ICDAS. This was a potential source of error. However, there was a high degree of equivalence in the detection of obvious decay between the examiners, suggesting that the oral health findings were trustworthy. Finally, the OHIP scores were not known before the people in custody had or had not accessed prison dental services and is, therefore, a potential limitation. Thus, although the findings of this work must be interpreted with caution, they nonetheless highlight the importance of accessibility factors and, in particular, health literacy and irritability with others as additional and intervening factors in reported access to prison dental services.

This survey identified and characterized access to prison dental services during custodial sentences. Of particular interest is the proposition that when oral health impacts quality of life, there appears to be increased awareness of the need for dental health treatment, which, in turn, promotes dental services’ attendance. These findings should be of interest to all those who work within the prison sector [31]. Allen et al. [32] have proposed the need to have ‘cross-sector collaborations’ when providing health care to reduce health inequity. This type of cross-sector collaboration is now in existence in Scotland in the form of the oral health promotion intervention for people in custody through a program called ‘Mouth Matters’. Mouth Matters represents a cross-sector collaboration between the Scottish Health Boards and the Scottish Prison Service [33]. This investigation provides additional support for ‘cross-sector collaboration’ and the need to work in partnership with those from the prison services, health-care colleagues, and those in custody to improve the oral health, health literacy, and the oral health-related quality of life of people in prison.

## 5. Conclusions

In conclusion, this investigation identified factors associated with access to prison dental services in Scotland. The role of accessibility factors, such as the oral health impact of irritability, appeared to increase perceptions of dental need and promote the attendance of dental services.

## Figures and Tables

**Table 1:** Comparison of Oral Health Impact Scale (OHIP) items by access to prison dental services.

Oral Health-Related Quality of Life	Accessed Prison Dental Services		t	P
OHIP Item	Yes (n = 118) x (SD)	No (n = 141) x (SD)		
uncomfortable eating	1.26 (1.33)	1.69 (1.50)	2.40	0.02
self-conscious about appearance	1.47 (1.52)	2.09 (1.59)	3.20	0.002
feeling tense about appearance	1.31 (1.40)	1.76 (1.53)	2.34	0.02
unsatisfactory diet	0.60 (1.00)	0.99 (1.34)	2.60	0.01
interrupted meals	0.76 (1.17)	1.26 (1.38)	3.27	0.001
difficulty relaxing	1.05 (1.29)	1.48 (1.42)	2.50	0.01
embarrassed about appearance	1.44 (1.57)	1.94 (1.66)	2.46	0.01
irritable with others	0.83 (1.25)	1.34 (1.45)	2.98	0.003
difficulty doing usual jobs	0.51 (0.96)	0.88 (1.26)	2.47	0.01
feeling unable to function	0.54 (1.03)	0.85 (1.26)	2.11	0.04

**Table 2:** Mean score of D_3cv_MFT, decayed, missing, and filled teeth in this Scottish prisoner population.

Accessed Dental Services in Prison	Decayed Teeth Mean (SD)	Missing Teeth Mean (SD)	Filled Teeth Mean (SD)	D_3cv_MFT Mean (SD)
Yes (n = 118)	1.36 (2.10)	5.93 (4.91)	4.93 (3.69)	12.22 (6.39)
No (n = 141)	1.84 (2.30)	3.05 (3.55)	3.65 (3.80)	8.54 (5.76)
t	1.78	5.32	2.75	4.82
P	0.08	<0.001	0.006	<0.001

**Table 3:** Identifying factors associated with access to prison dental services.

Factors Associated with Accessing Services	Model 1			Model 2			Model 3		
	OR	95 %CI	P	OR	95% CI	P	OR	95% CI	P
Age (in years)	1.05	1.01, 1.09	<0.006	1.05	1.02, 1.09	<0.004	1.03	0.99, 1.07	0.20
Prison experience (number of times in remand)	1.06	1.00, 1.14	0.04	1.05	0.99, 1.12	0.004	1.08	1.01, 1.16	0.03
OHIP: irritable with people				1.36	1.11, 1.66	0.003	1.32	1.06, 1.66	0.02
Pattern of dental attendance outside of prison: routine emergency							1 2.17	1.19, 3.93	0.01
Total number of decayed teeth (D3_cv_T)							0.82	0.70, 0.95	0.01
Total number of missing teeth (MT)							1.13	1.04, 1.22	0.004
Variance explained	19%			26%			35%		
−2 log likelihood	164.89			151.97			147.68		
df	3			4			7		
∆X^2^ (∆df)				9.30 (1)			20.58 (3)		
P				0.002			<0.001		

Model 1: Adjusted for age and prison remands. Model 2: Adjusted for variables in Model 1 plus OHIP item ‘feeling irritable with people because of teeth, mouth, or dentures’. Model 3: Adjusted for variable in Model 2 plus pattern of dental attendance outside of prison, total number of D_3cv_T, and total number of MT.

## References

[1] Heidari E., Dickson C., Newton T. (2014). An overview of the prison population and the general health status of prisoners. Br. Dent. J..

[2] Akaji E., Ashiwaju M. (2014). Oral health status of a sample of prisoners in Enugu: A disadvantaged population. Ann. Med. Health Sci. Res..

[3] Osborn M., Butler T., Barnard P.D. (2003). Oral health status of prison inmates—New South Wales, Australia. Aust. Dent. J..

[4] Guarnizo-Herreño C.C., Watt R.G., Fuller E., Steele J.G., Shen J., Morris S., Wildman J., Tsakos G. (2014). Socioeconomic position and subjective oral health: Findings for the adult population in England, Wales and Northern Ireland. BMC Public Health.

[5] Jones C., McCann M., Nugent Z. Scottish Prisons Dental Health Survey 2002. http://www.scotland.gov.uk/Resource/Doc/47210/0013527.pdf.

[6] Heidari E., Dickinson C., Wilson R., Fiske J. (2007). Oral health of remand prisoners in HMP Brixton, London. Br. Dent. J..

[7] Rodrigues I.S., Silveira I.T., Pinto M.S., Xavier A.F., de Oliveira T.B., de Paiva S.M., de Castro R.D., Cavalcanti A.L. (2014). Locked mouths: Tooth loss in a women’s prison in northeastern Brazil. Sci. World J..

[8] World Health Organization Europe Guide on Prison and Health. http://www.euro.who.int/en/health-topics/health-determinants/prisons-andhealth/publications/2014/prisons-and-health.

[9] Brutus L., Mackie P., Millard A., Fraser A., Conacher A., Hardie S., McDowall L., Meechan H. Better Health, Better Lives for Prisoners: A Framework for Improving the Health of Scotland’s Prisoners: Volume 1: The Framework. ScotPHN, Glasgow, 2012. http://www.scotphn.net/pdf/2012_06_08_Health_improvement_for_prisoners_vol_1_Final_.

[10] Everington T. Healthier People Safer Communities: Working Together to Improve Outcomes for Offenders. https://www.scotphn.net/wp-content/uploads/2015/10/Healthier-People-Safer-Communities-April-2013.pdf.

[11] Freeman R., Akbar T., Buls D., Edwards M., Everington T., Richards D., Themessl-Huber M., Watt C. The Oral Health and Psychosocial Needs of Scottish Prisoners and Young Offenders. http://dentistry.dundee.ac.uk/sites/dentistry.dundee.ac.uk/files/media/SOHIPP-report.pdf.

[12] Donelle L., Hall J. (2014). An exploration of women offenders’ health literacy. Soc. Work Public Health.

[13] Heidari E., Dickinson C., Newton T. (2014). Multidisciplinary team working in an adult male prison establishment in the UK. Br. Dent. J..

[14] Buunk-Werkhoven Y.A., Dijkstra A., Schaub R.M., van der Schans C.P., Spreen M. (2010). Oral health related quality of life among imprisoned Dutch forensic psychiatric patients. J. Forensic. Nurs..

[15] Vacca J.S. (2004). Educated prisoners are less likely to return to prison. J. Correct. Educ..

[16] Cohen L.K. (1987). Converting unmet need for care to effective demand. Int. Dent. J..

[17] Marshman Z., Porritt J., Dyer T., Wyborn C., Godson J., Baker S. (2012). What influences the use of dental services by adults in the UK?. Community Dent. Oral Epidemiol..

[18] Marshman Z., Baker S.R., Robinson P.G. (2014). Does dental indifference influence the oral health-related quality of life of prisoners?. Community Dent. Oral Epidemiol..

[19] Smith P.A., Themessl-Huber M., Akbar T., Richards D., Freeman R. (2011). What motivates dentists to work in prisons? A qualitative exploration. Br. Dent. J..

[20] Gillett R. (1994). Post hoc power analysis. J. Appl. Psychol..

[21] Hill K.B., Chadwick B., Freeman R., O’Sullivan I., Murray J.J. (2013). Adult Dental Health Survey 2009: Relationships between dental attendance patterns, oral health behaviour and the current barriers to dental care. Br. Dent. J..

[22] Humphris G.M., Crawford J.R., Hill K., Gilbert A., Freeman R. (2013). UK Population Norms for the Modified Dental Anxiety Scale with Percentile Calculator: Adult Dental Health Survey 2009 Results. BMC Oral Health.

[23] Newton J.T., Edwards J.C. (2005). Psychometric properties of the modified dental anxiety scale: An independent replication. Community Dent. Health.

[24] Locker D., Allen P.F. (2002). Developing short-form measures of oral health-related quality of life. J. Public Health Dent..

[25] Robinson P.G., Gibson B., Khan F.A., Birnbaum W. (2003). Validity of two oral health-related quality of life measures. Community Dent. Oral Epidemiol..

[26] Scottish Government Prison Statistics and Population Projections Scotland: 2011-12. http://www.scotland.gov.uk/Resource/0039/00396363.pdf.

[27] NHS National Services Scotland National Dental Inspection Programme. http://www.isdscotland.org/Health-Topics/Dental-Care/National-Dental-Inspection-Programme/.

[28] Ismail A.I., Sohn W., Tellez M., Amaya A., Sen A., Hasson H., Pitts N.B. (2007). The International Caries Detection and Assessment System (ICDAS): An Integrated System for Measuring Dental Caries. Community Dent. Oral Epidemiol..

[29] ICDAS Foundation International Caries Detection and Assessment System. https://www.icdas.org/.

[30] International Caries Detection and Assessment System (ICDAS) Coordinating Committee Criteria Manual International Caries Detection and Assessment System. https://www.icdas.org/uploads/ICDAS%20Criteria%20Document%20corrected%202013.pdf.

[31] Costa J. (2014). Dental care in corrections. Dis. Mon..

[32] Allen J., Goldblatt P., Daly S., Jabbal J., Marmot M. (2018). Reducing Health Inequalities through New Models of Care: A Resource for New Care Models. http://www.instituteofhealthequity.org/resources-reports/reducing-health-inequalities-through-new-models-of-care-a-resource-for-new-care-models.

[33] NHS Health Scotland Mouth Matters Guide to Trainers: Better Oral Care for Offenders. http://www.knowledge.scot.nhs.uk/media/CLT/ResourceUploads/4086716/a2b802de-6f65-4594-b749-01cfe0f5b9e4.pdf.

